# Serum levels of soluble CD163 and CXCL5 may be predictive markers for immune-related adverse events in patients with advanced melanoma treated with nivolumab: a pilot study

**DOI:** 10.18632/oncotarget.24509

**Published:** 2018-02-15

**Authors:** Taku Fujimura, Yota Sato, Kayo Tanita, Yumi Kambayashi, Atsushi Otsuka, Yasuhiro Fujisawa, Koji Yoshino, Shigeto Matsushita, Takeru Funakoshi, Hiroo Hata, Yuki Yamamoto, Hiroshi Uchi, Yumi Nonomura, Ryota Tanaka, Megumi Aoki, Keisuke Imafuku, Hisako Okuhira, Sadanori Furudate, Takanori Hidaka, Setsuya Aiba

**Affiliations:** ^1^ Department of Dermatology, Tohoku University Graduate School of Medicine, Sendai, Japan; ^2^ Department of Dermatology, Kyoto University Graduate School of Medicine, Kyoto, Japan; ^3^ Department of Dermatology, Faculty of University of Tsukuba, Tsukuba, Japan; ^4^ Department of Dermatology, Tokyo Metropolitan Cancer and Infectious Disease Center Komagome Hospital, Tokyo, Japan; ^5^ Department of Dermato-Oncology/Dermatology, National Hospital Organization Kagoshima Medical Center, Kagoshima, Japan; ^6^ Department of Dermatology, Keio University School of Medicine, Tokyo, Japan; ^7^ Department of Dermatology, Hokkaido University Graduate School of Medicine, Sapporo, Japan; ^8^ Department of Dermatology, Wakayama Medical University, Wakayama, Japan; ^9^ Department of Dermatology, Kyushu University Graduate School of Medicine, Fukuoka, Japan

**Keywords:** sCD163, CXCL5, TAMs, melanoma, POSTN

## Abstract

Antibodies against PD-1, such as nivolumab and pembrolizumab, are widely used in the treatment of various cancers including advanced melanoma. The anti-PD-1 Ab significantly prolongs survival in patients with metastatic melanoma, and its administration in combination with local or systemic therapy may also lead to improved outcomes. Although anti-PD-1 Ab-based combined therapy might be effective for the treatment of advanced melanoma, the associated risk of irAEs is an important consideration. Therefore, being able to predict irAEs is of great interest to oncologists. The purpose of this study was to evaluate the value of using serum levels of sCD163 and CXCL5 to predict irAEs in patients with advanced melanoma who were administered nivolumab. To this end, we analyzed these serum levels in 46 cases of advanced melanoma treated with nivolumab. In addition, the tumor stroma was evaluated by immunohistochemistry and immunofluorescence. We measured the serum levels of sCD163 and CXCL5 on day 0 (immediately before nivolumab administration) and day 42. The serum absolute levels of sCD163 were significantly increased in patients who developed AEs (*p* = 0.0018). Although there was no significant difference in serum levels of CXCL5, the absolute value of CXCL5 could at least be a supportive marker for the increased absolute levels of serum sCD163. This study suggests that sCD163 and CXCL5 may serve as possible prognostic biomarkers for irAEs in patients with advanced melanoma treated with nivolumab.

## INTRODUCTION

The program cell death-1/programmed death-ligand 1 (PD-1/PD-L1) pathway plays a critical role in the tumor immune response; thus, anti-PD-1 antibodies (Abs), such as nivolumab and pembrolizumab, are widely used in the treatment of various cancers including advanced melanoma [1–3]. The anti-PD-1 Ab significantly prolongs survival in patients with metastatic melanoma, and its administration in combination with local [4–8] or systemic therapy (e.g., ipilimumab, epacadostat) [2, 9] may also lead to improved outcomes. Although anti-PD-1 Ab-based combined therapy might prove effective for the treatment of advanced melanoma, the associated risk of immune-related adverse events (irAEs) such as severe hepatitis, interstitial pneumonia, colitis, type 1 diabetes mellitus, hypophysitis, or myasthenia gravis is an important consideration [2]. Therefore, the prediction of irAEs is of great interest among oncologists.

Tumor-associated macrophages (TAMs) are characterized by their heterogeneity and plasticity, and may be functionally reprogrammed to polarized phenotypes by exposure to cancer-related factors, stromal factors, or infection, leading to the production of various chemokines that are specific to each cancer [10–14]. Concerning metastatic melanoma, periostin (POSTN) was found to be expressed in the region surrounding melanoma cell nests in the metastatic melanoma lesions of wounded mice and humans [15], suggesting that it might stimulate TAMs to produce chemokines that induce melanoma-specific tumor-infiltrating lymphocytes (TILs) in melanoma patients with systemic inflammation. As we previously reported, upon POSTN stimulation, M2 macrophages produce several immunosuppressive and autoimmune-related chemokines including CXCL5 [14]. Notably, CXCL5 is a biomarker of T helper 17 cell-mediated autoimmune diseases such as multiple sclerosis, rheumatoid arthritis, and pemphigus vulgaris [16–18]; and soluble CD163 (sCD163) is a TAM marker that appears in the serum as a result of proteolytic shedding [19]. We previously reported that both CXCL5 and sCD163 might predict irAEs in 17 patients with advanced melanoma patients treated with nivolumab [8, 20]. In this report, we further analyzed the serum levels of sCD163 and CXCL5 in 46 cases of advanced melanoma treated with nivolumab.

## RESULTS

### Patients

We collected data from 46 patients treated with nivolumab (Table 1). The mean patient age was 66.5 years (range, 31–93 years) and 52.8% were males. The most common site of the primary tumor was in the extremities (39.1%), followed by mucosal origin (30.4%), trunk (15.2%), head/neck (6.5%), and the ocular region (2.2%). A dose of 2 mg/kg nivolumab was administered to the patients followed by 3 weeks of rest. The incidence of AE development was 45.7% (Grade 4: 6.5%, Grade 3: 15.2%, Grade 2: 19.6%, Grade 1: 6.5%), and the average onset of irAE development was 136 days (range, 21–370).

**Table 1:** Patient characteristics and serum levels of sCD163 and CXCL5

	Age	Sex	Onset of irAE	Location	sCD163 (ng/ml)	CXCL5 (pg/ml)	irAE	Grade
1	68	M	237	Extremities	115.782943	35.56162835	hypophisitis	4
2	36	M	55	Extremities	–44.23121476	–17.57753725	hepatitis	4
3	61	M	245	Extremities	–11.4747151	174.0938997	interstitial pneumonia	4
4	83	F	370	Extremities	40.72768869	–31.97872991	radiation dermatitis	3
5	85	F	27	Other/Unknown	–25.44524995	–91.17233749	chronic inflammatory demyelinating polyneuropathy	3
6	66	F	214	Ocular	43.18056154	–22.79478559	rheumarthritis	3
7	34	M	84	Trunk	–19.92970319	61.91409025	biliary tract disorder	3
8	68	M	60	Extremities	36.99828277	–27.63658318	bursitis	3
9	77	M	346	Trunk	21.42662403	–54.81110386	psoriasiform dermatitis	3
10	67	F	23	Mucosa	20.55968805	–15.27295948	psoriasiform dermatitis	3
11	75	F	42	Mucosa	140.8945355	–1.171878513	interstitial pneumonia	2
12	54	F	126	Mucosa	3.937154859	–15.87606189	diarrhea	2
13	62	F	121	Trunk	–36.4146062	–48.4018883	adrenal insufficiency	2
14	81	F	77	Extremities	–22.47729919	–70.94219363	thyroid dysfunction	2
15	67	M	132	Extremities	34.16606428	6.260637076	hypophisitis	2
16	61	F	21	Extremities	–8.525671721	–3.390285544	thyroid dysfunction	2
17	65	M	221	Extremities	–76.45520955	2.912773985	thyroid dysfunction	2
18	69	F	62	Mucosa	–33.29336941	25.19612409	thyroid dysfunction	2
19	76	F	210	Extremities	28.36775442	103.3236074	hypophisitis	2
20	33	F	84	Extremities	77.54691463	0.457742932	hypophisitis	1
21	77	F	143	Mucosa	–41.53950593	–24.59772023	adrenal insufficiency	1
22	62	M	92	Other/Unknown	–47.99843144	–11.35075991	adrenal insufficiency	1
23	60	M	N.A.	Trunk	–4.542122094	–16.16551984	N.A.	N.A.
24	34	F	N.A.	Extremities	–29.55718265	0.254223734	N.A.	N.A.
25	61	F	N.A.	Mucosa	–47.76858108	–80.42942923	N.A.	N.A.
26	76	M	N.A.	Trunk	–26.66666667	–19.75628258	N.A.	N.A.
27	79	M	N.A.	Head/Neck	–8.395159834	–18.21571007	N.A.	N.A.
28	84	F	N.A.	Trunk	4.932359223	–2.175956805	N.A.	N.A.
29	68	M	N.A.	Extremities	14.99180027	–24.6852751	N.A.	N.A.
30	93	M	N.A.	Extremities	1.939261048	2.454790431	N.A.	N.A.
31	74	M	N.A.	Trunk	–22.07524444	1.421405214	N.A.	N.A.
32	54	M	N.A.	Mucosa	–4.745006346	–10.67711099	N.A.	N.A.
33	67	M	N.A.	Extremities	3.416168176	–40.954426	N.A.	N.A.
34	70	M	N.A.	Head/Neck	–12.31934772	–26.09262273	N.A.	N.A.
35	65	M	N.A.	Mucosa	11.10220568	1.102732526	N.A.	N.A.
36	79	F	N.A.	Extremities	–30.35604275	14.93305921	N.A.	N.A.
37	72	M	N.A.	Mucosa	–4.40651476	194.1480243	N.A.	N.A.
38	89	M	N.A.	Other/Unknown	–0.721084093	–26.81119932	N.A.	N.A.
39	55	F	N.A.	Mucosa	3.390383922	44.79528142	N.A.	N.A.
40	82	M	N.A.	Mucosa	46.17573498	33.3969329	N.A.	N.A.
41	73	M	N.A.	Extremities	–19.03589077	–7.105634886	N.A.	N.A.
42	62	F	N.A.	Trunk	–58.26412844	31.31318466	N.A.	N.A.
43	31	F	N.A.	Head/Neck	–15.77316618	9.483456384	N.A.	N.A.
44	64	M	N.A.	Mucosa	8.078227869	20.27538744	N.A.	N.A.
45	77	F	N.A.	Mucosa	–42.70869091	–27.32383979	N.A.	N.A.
46	65	F	N.A.	Extremities	–16.12972729	–24.15391828	N.A.	N.A.

Serum levels of sCD163 and CXCL5 from each patient (*n* = 46) on days 0 and 42 were examined by ELISA.

### Serum levels of sCD163 and CXCL5

**Figure 1:**
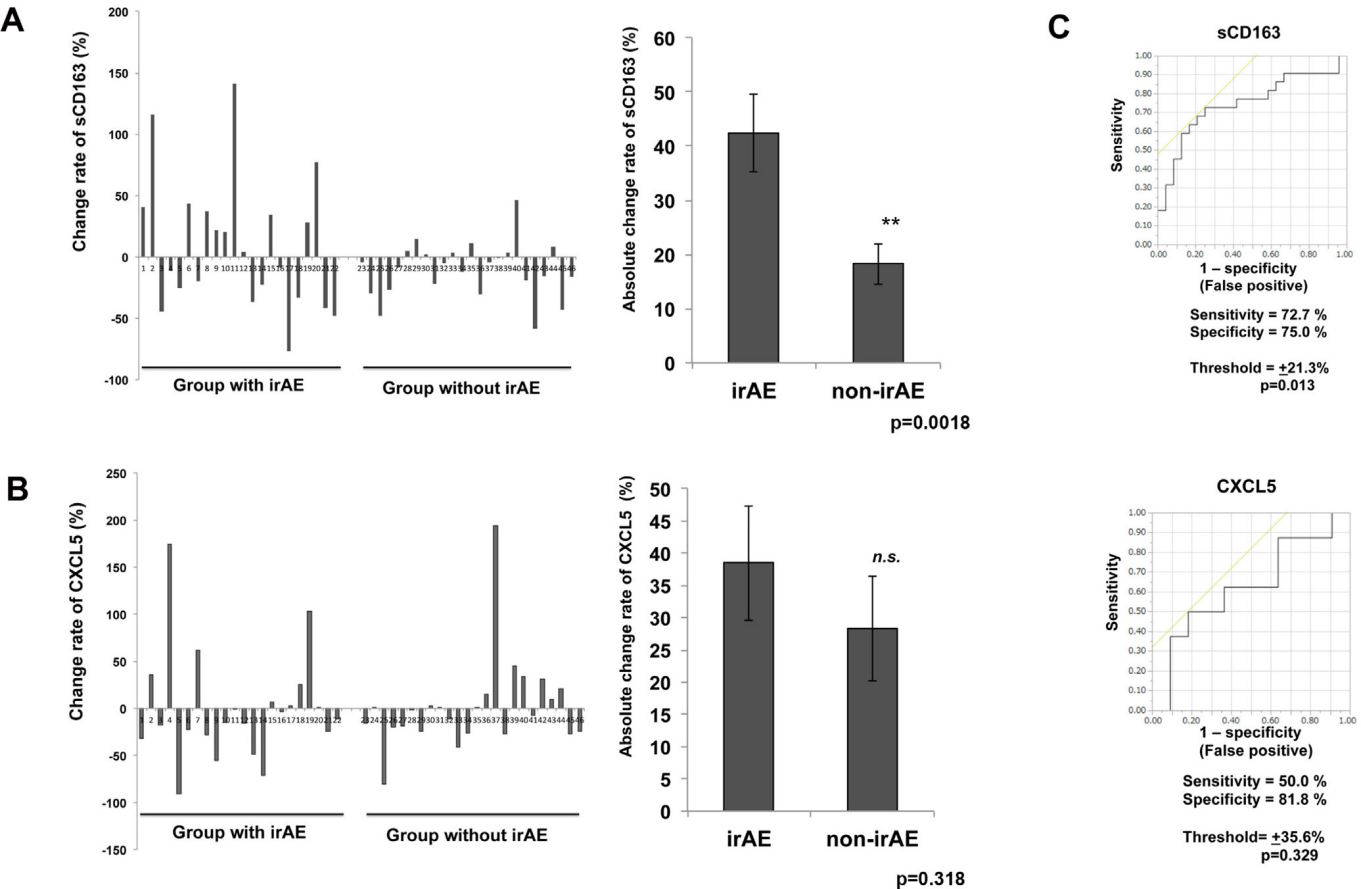
ROC curve of serum levels of sCD163 or CXCL5 The change in serum levels of sCD163 (**A**) and CXCL5 (**B**) from each patient (*n* = 46) with or without irAE on day 42. The ROC curve was applied to calculate the cut-off values of the sCD163 or CXCL5 serum levels and AUC (**C**). The cut-off point was determined using Youden’s index.

To determine whether serum levels of sCD163 and CXCL5 may predict AEs in patients treated with nivolumab, we evaluated their levels in 46 patients with advanced melanoma treated with nivolumab. Compared to baseline (day 0), the absolute value of sCD163 serum levels at day 42 were significantly increased in the group that developed irAEs compared with the group lacking irAEs (*p* = 0.0018; Figure 1A), whereas there was no significant difference in the absolute value of serum CXCL5 (Figure 1B). The increase or decrease of serum sCD163 and CXCL5 in each patient (Table 1) and each irAE (Table 2) is described. The cut-off point was determined using Youden’s index. The threshold of the change rate of serum sCD163 was ± 21.3%, while the threshold of the change rate of serum CXCL5 was ± 35.6%. The sensitivity of serum sCD163 was 72.7%, whereas that of CXCL5 was 50%. The specificity of serum sCD163 was 75.0%, whereas that of CXCL5 was 81.8% (Figure 1C).

**Table 2:** Serum levels of sCD163 and CXCL5 in each irAE

Case	irAE	Grade	sCD163 (ng/ml)	sCD163	CXCL5 (pg/ml)	CXCL5
1	hypophisitis	4	115.782943	+	35.56162835	+
15	hypophisitis	2	34.16606428	+	6.260637076	+
19	hypophisitis	2	28.36775442	+	103.3236074	+
20	hypophisitis	1	77.54691463	+	0.457742932	+
14	thyroid dysfunction	2	−22.47729919	−	−70.94219363	−
16	thyroid dysfunction	2	−8.525671721	−	−3.390285544	−
17	thyroid dysfunction	2	−76.45520955	−	2.912773985	+
18	thyroid dysfunction	2	−33.29336941	−	25.19612409	+
13	adrenal insufficiency	2	−36.4146062	−	−48.4018883	−
21	adrenal insufficiency	1	−41.53950593	−	−24.59772023	−
22	adrenal insufficiency	1	−47.99843144	−	−11.35075991	−
9	psoriasiform dermatitis	3	21.42662403	+	−54.81110386	−
10	psoriasiform dermatitis	3	20.55968805	+	−15.27295948	−
3	interstitial pneumonia	4	−11.4747151	−	174.0938997	+
11	interstitial pneumonia	2	140.8945355	+	−1.171878513	−
2	hepatitis	4	−44.23121476	−	−17.57753725	−
4	radiation dermatitis	3	40.72768869	−	−31.97872991	−
5	chronic inflammatory demyelinating polyneuropathy	3	−25.44524995	−	−91.17233749	−
6	rheumarthritis	3	43.18056154	−	−22.79478559	−
7	biliary tract disorder	3	−19.92970319	+	61.91409025	+
8	bursitis	3	36.99828277	−	−27.63658318	−

Serum levels of sCD163 and CXCL5 from each patient (*n* = 46) on days 0 and 42 were examined by ELISA in each irAE.

### Tissue samples, immunohistochemistry, and immunofluorescence

**Figure 2:**
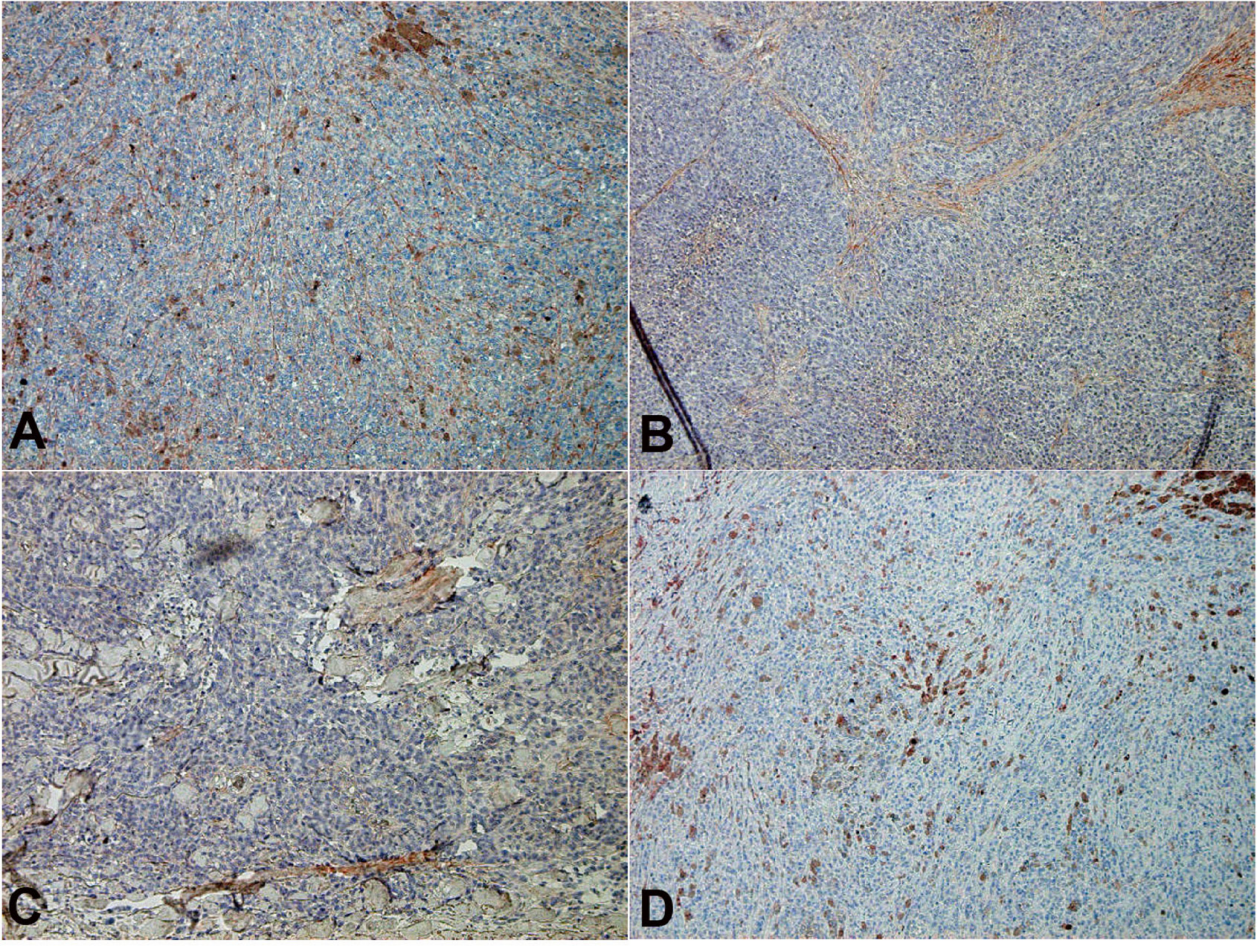
POSTN expression in in-transit melanoma Sections of in-transit melanoma from patients with irAE after nivolumab (**A**) and before nivolumab administration (**B**), and patients without irAE (**C**) were deparaffinized and stained using anti-POSTN (A, B, C) or anti-CD163 Abs (**D**). The signal was developed with 3-amino-9-ethylcarbazole.

**Figure 3:**
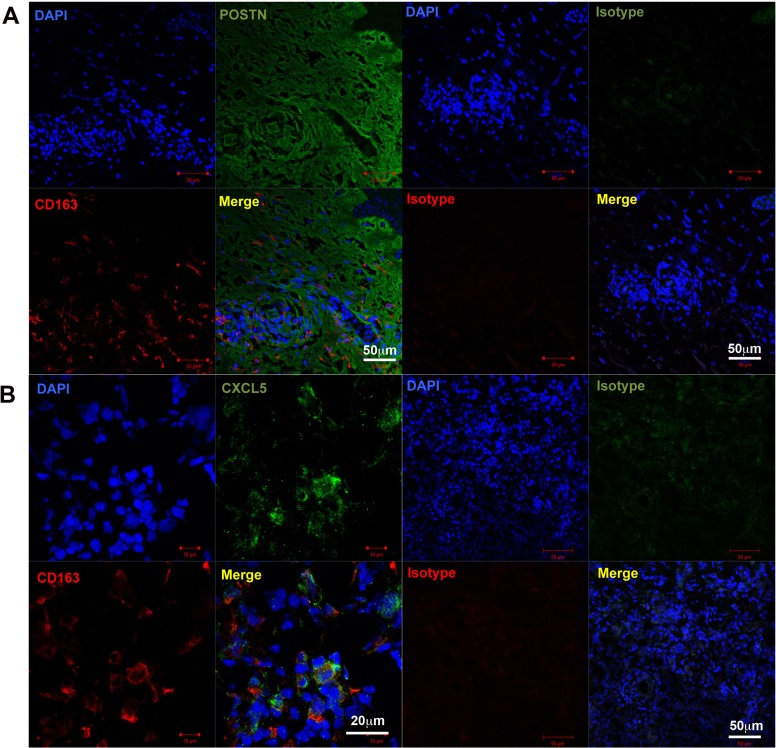
CD163+ cells in in-transit melanoma Immunofluorescence staining of in-transit melanoma for (**A**) POSTN (green), CD163 (red), and DAPI (blue, nucleus), and (**B**) CXCL5 (green), CD163 (red), and DAPI (blue, nucleus). A merged image is also shown. Merged green and red stain yellow. Representative specimens from five cases are shown.

Because sCD163 is an activation marker for CD163^+^ tissue macrophages [27], CXCL5 is produced from CD163+ macrophages by POSTN stimulation [14], and POSTN in melanoma is augmented by nonspecific inflammation such as wound healing [15], we hypothesized that the administration of nivolumab in advanced melanoma might increase the expression of POSTN in melanoma tissue, leading stimulated CD163 macrophages to produce sCD163 and CXCL5. To prove our hypothesis, we performed immunohistochemical staining for POSTN and CD163 in five patients with in-transit advanced melanoma without irAEs, and in two patients with in-transit advanced melanoma with irAEs. The expression of POSTN in in-transit melanoma was augmented in stromal fibroblasts in patients with irAE, especially after nivolumab administration (Figure 2A). In contrast, the expression of POSTN in in-transit melanoma from irAE-developed patients before administration of nivolumab (Figure 2B) and in-transit melanoma from non-irAE-developed patients (Figure 2C) was low in fibroblasts distributed in peritumoral lesions. The semi-quantitative analysis of POSTN expression is described in Table 3. CD163+ cells were detected in each group (Figure 2D). The number of CD163+ cells significantly increased in patients with irAEs (Supplementary Figure 1). Notably, immunofluorescence staining revealed that CD163+ macrophages were distributed in POSTN-expressing areas (Figure 3A), suggesting that POSTN in melanoma can stimulate CD163+ macrophages to produce CXCL5 (Figure 3B).

**Table 3:** Semi-quantitative analysis of immunohistochemical staining of POSTN

	irAE grade	Pre treatment	Post treatment
**Case 1**	**4**	**+**	**+++**
**Case 4**	**3**	**+**	**+++**
**Case 23**	**0**	**++**	**+**
**Case 28**	**0**	**++**	**+**
**Case 41**	**0**	**+**	**+**
		**-**	**negative**
		**+**	**weak**
		**++**	**moderate**
		**+++**	**intense**

The intensity of immunohistochemical staining of POSTN was scored on a semi-quantitative scale.

## DISCUSSION

Because of nivolumab’s higher efficacy than other anti-melanoma drugs (e.g., ipilimumab and dacarbazine) [1, 21], and because it induces a longer duration of anti-tumor response than BRAF/MEK inhibitors (e.g., vemurafenib, dabrafenib, and trametinib) [22, 23], oncologists have been particularly interested in combining nivolumab with agents that enhance the anti-tumor immune response in patients with metastatic melanoma [3, 8, 24, 25]. The efficacy of nivolumab is significantly increased when combined with ipilimumab (57.7%); however, unfortunately, the rate of severe treatment-related AEs (Grade 3 or 4) is also significantly increased with this particular combination (55.0%) [2, 24]. These findings suggest the importance of predicting irAEs to avoid severe complications using simple methods. Indeed, Fujisawa *et al.* [26] reported that the decrease of lymphocytes fraction could predict irAEs caused by nivolumab, but only in the very short period before irAE development. Since the interval for nivolumab administration is 2 or 3 weeks for melanoma patients, other prognostic biomarkers are needed for long intervals.

CD163 is a member of the scavenger receptor cysteine-rich family, and is exclusively expressed on cells of monocyte/macrophage lineage [19, 27]. In lesions of various skin cancers, CD163+ macrophages are a main component of TILs that can produce various chemokines by stimulating cancer-specific stromal factors such as POSTN, IL-4, and RANKL [11, 14, 28]. sCD163 is an activation marker for CD163^+^ tissue macrophages that is present in the serum as a result of proteolytic shedding [19]. Notably, serum sCD163 levels increase in autoimmune diseases such as atherosclerosis, rheumatoid arthritis, pemphigus vulgaris, and bullous pemphigoid [17, 19, 28], and reflect disease activity [19]. In aggregate, sCD163 could be a macrophage activation marker to predict the production of cancer-specific chemokines from TAMs in different skin cancers.

CXCL5 is a chemokine that can recruit neutrophils and CXCR2+ myeloid cells, including myeloid-derived suppressor cells (MDSCs) and monocytes that can be a precursor of TAMs in tumor-bearing hosts [29]. Recently, Katoh *et al.* [29] reported that the inhibition of CXCR2+ MDSC recruitment into colonic mucosa and tumors dramatically suppress colonic inflammation, suggesting that CXCR2+ MDSCs are essential for colitis-associated cancer. In another report, Steele *et al.* [30] suggested that CXCR2 signaling in the myeloid component can promote pancreatic tumorigenesis and is required for pancreatic cancer metastasis. Zhang *et al.* [31] reported that the depletion of MDSCs decreased IL-17A and IL-1β production, leading to therapeutic effects of arthritis in a mouse model, suggesting the importance of pro-inflammatory MDSCs in autoimmune disease. MDSCs are a heterogeneous population of cells that can be induced by tumor-associated inflammation, including autoimmune related cytokines such as IL-1β and IL-6 [32]. Because POSTN is an extracellular matrix protein that can be detected in the dermis of various skin inflammatory disorders including autoimmune disease such as scleroderma, pemphigus vulgaris, and bullous pemphigoid [17, 33], POSTN in melanoma might stimulate MDSCs to further produce chemokines such as CXCL5.

In this report, we analyzed the serum levels of sCD163 and CXCL5 in 46 cases of advanced melanoma treated with nivolumab. The tumor stroma of melanoma had increased expression of POSTN upon administration with nivolumab, and POSTN stimulated TAMs to release CXCL5 [14]. The increased absolute value of serum levels of sCD163 and CXCL5 in a patient who develops adverse nivolumab-induced, immune-related events is presumably related to POSTN-stimulated TAM activation. Therefore, in the setting of nivolumab therapy, sCD163 and CXCL5 serum levels may serve as valuable predictors of irAEs [8, 20]; thus, we measured serum levels of sCD163 and CXCL5 at day 0 (immediately before nivolumab administration) and day 42. In addition, as presented in Table 2, the change in levels of sCD163 and CXCL5 tend to differ in each irAE. Although we could not determine the precise reasons, one possible explanation of this observation is that the time point of TAM activation differs in each irAE. Because the recruitment of effector cells to target organs is indispensable for the development of irAEs, and since one of the main sources of chemokines are macrophages, the activation of macrophages is important for the development of irAEs. Decreased serum levels of sCD163, especially in cases of thyroid dysfunction or adrenal insufficiency, indicates that CD163+ macrophages have been activated and effector cells have been recruited to the target organ, both of which underly the development of irAEs by nivolumab. Therefore, serum levels of sCD163 were significantly increased or decreased in patients who developed AEs (*p* = 0.013). This phenomenon could be explained by the heterogeneity of TAMs, which consist of macrophages in various phases of activation [34]. Indeed, the serum levels of CXCL5 correlate with autoimmune diseases such as multiple sclerosis, rheumatoid arthritis, glomerulonephritis, pemphigus vulgaris, and bullous pemphigoid with several inflammation factors [16–18, 35], though the main sources of CXCL5 is not fully investigated in each reports. Thus, although there was no significant difference in CXCL5 levels, the change rate of CXCL5 may be a useful marker for predicting the organs affected by irAEs. Because this was a pilot study, future independent studies with a larger patient cohort are needed to confirm our findings.

### Ethics statement for animal and human experiments

The protocol for the human study was approved by the ethics committee of Tohoku University Graduate School of Medicine, Sendai, Japan (Permit No: 2017-1-377).

## PATIENTS AND METHODS

Data from patients who were treated with nivolumab were collected from six clinical sites in Japan. Patients were eligible if they had unresectable stage III melanoma, if their tumor was resectable but they had declined resection, or if they had stage IV melanoma with accessible cutaneous, subcutaneous, and/or nodal lesions (patients were staged according to the AJCC Staging Manual, 7th Edition, 2011). All patients received 2 mg/kg nivolumab followed by a 3-week rest period or 3 mg/kg nivolumab followed by 2 weeks of rest, both of which are the approved dosing schedule in Japan. Serum from patients was obtained on days 0 and 42. In our 46 cases, case 5 was administered systemic steroid on day 28, and the serum levels in this case may have been affected by systemic steroid. The other two cases (cases 10 and 16) were treated with topical steroid (case 10) or levothyroxine sodium hydrate (case 16).

### Serum levels of sCD163 and CXCL5

On days 0 and 42 after nivolumab administration, we analyzed the serum levels of soluble sCD163 and CXCL5 by enzyme-linked immunoassay (ELISA) according to the manufacturer’s protocol (R&D Systems, Minneapolis, MN, USA). The serum levels on day 42 were compared to baseline (day 0) and statistically analyzed.

### Reagents

The following Abs were used for immunohistochemistry: mouse anti-human CD163 monoclonal (Novocastra, Tokyo, Japan) and rabbit polyclonal anti-POSTN (Abcam, Tokyo, Japan). The following Abs were used for immunofluorescence: mouse anti-human CD163 phycoerythrin-conjugated monoclonal antibody (R&D Systems), rabbit polyclonal POSTN (Abcam), and Alexa Fluor 488-conjugated anti-rabbit goat IgG (Abcam).

### Tissue samples, immunohistochemistry, and immunofluorescence

Archival formalin-fixed paraffin-embedded skin specimens were collected from five patients with in-transit advanced melanoma without irAEs, and from two patients with in-transit advanced melanoma with irAEs (Grades 2 and 3 hypophysitis), who were treated in the Department of Dermatology at Tohoku University Graduate School of Medicine. All patients gave written informed consent. The in-transit melanoma samples were processed for single staining of CD163a and POSTN, and the signal was developed with 3-amino-9-ethylcarbazole (Nichirei Bioscience, Tokyo, Japan). For cryosections, five cases of in-transit melanoma samples were frozen in optimal cutting temperature embedding medium (Sakura Finetek Japan Co. Ltd., Tokyo, Japan), and 6 μm thick sections were fixed in cold acetone for 30 min and blocked in immunofluorescence buffer (phosphate-buffered saline, 5% bovine serum albumin). Thereafter, each section was incubated with the relevant antibodies. The slides were mounted in DAPI Fluoromount-G (Southern Biotech, Birmingham, AL, USA) and examined using a Zeiss LSM 700 microscope equipped with a Spot digital camera.

### Statistical methods

The receiver operating characteristic (ROC) curve was applied to calculate the cut-off values for the serum levels of sCD163 or CXCL5 and the area under the curve (AUC). The cut-off point was determined using Youden’s index. The ROC curves were established to evaluate the serum levels of sCD163 and CXCL5 in patients who developed irAEs. For a single comparison between two groups, the Mann–Whitney *U*-test or Student’s *t*-test was used. Correlation coefficients were determined using the Spearman’s rank correlation test. The level of significance was set at *p* < 0.05. All statistical analyses were performed using JMP 13.1 software (SAS Institute Inc., Tokyo, Japan).

## SUPPLEMENTARY MATERIALS FIGURE


