# Supplementary material for: Serum levels of soluble CD163 and CXCL5 may be predictive markers for immune-related adverse events in patients with advanced melanoma treated with nivolumab: a pilot study
Source: Oncotarget. 2018 Feb 15;9(21):15542–51. doi: 10.18632/oncotarget.24509 (PMC5884646; doi:10.18632/oncotarget.24509)
Supplement: Supplementary file 1 [file oncotarget-09-15542-s001.pdf]

Serum levels of soluble CD163 and CXCL5 may be predictive markers for immune-related adverse events in patients with advanced melanoma treated with nivolumab: a pilot study

SUPPLEMENTARY MATERIALS

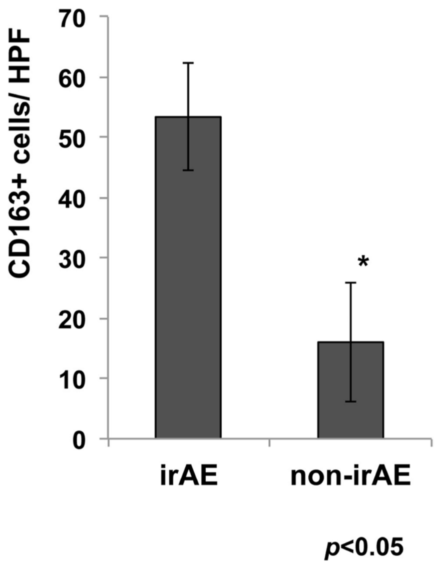

Supplementary Figure 1: The number of CD163+ cells in in-transit melanoma. The number of immunoreactive cells was counted using an ocular grid of 1 cm^2 at a magnification of 400x. The data are expressed as the means ± SDs of the numbers in each area. For a single comparison of two groups, the Mann–Whitney *U*-test or Student's *t*-test was used ($p < 0.05$).
